# Correlation Between MRI and Histopathology in Assessing Treatment Response to Neoadjuvant Chemoradiation in Locally Advanced Rectal Cancer

**DOI:** 10.1002/cnr2.70322

**Published:** 2025-08-28

**Authors:** Pedram Fadavi, Kambiz Novin, Maryam Garousi, Amir Mohammad Arefpour, Seyedeh Shaghayegh Rezvani Nezhad, Mahshid Soori, Farzad Taghizadeh‐Hesary

**Affiliations:** ^1^ Department of Radiation Oncology School of Medicine, Iran University of Medical Sciences Tehran Iran; ^2^ Department of Radiation Oncology School of Medicine, Shahid Beheshti University of Medical Sciences Tehran Iran; ^3^ ENT and Head and Neck Research Center and Department, the Five Senses Health Institute, School of Medicine Iran University of Medical Sciences Tehran Iran

**Keywords:** chemoradiotherapy, magnetic resonance imaging, neoadjuvant therapy, pathology, rectal cancer

## Abstract

**Background and Aims:**

Recognizing the predictive factors of response to chemoradiation is of utmost importance in patients with locally advanced rectal cancer (LARC). The study is designed to explore the correlation between post‐neoadjuvant chemoradiation (nCRT) pathologic and MR‐based tumor staging in LARC.

**Methods:**

Patients with non‐metastatic LARC underwent contrast‐enhanced pelvic MRI before and after treatment, received standard chemoradiation, and were evaluated for tumor response. Tumor regression grade (TRG) based on histopathology ranged from TRG0 to TRG3, while MR‐based responses encompassed complete response, partial response, stable disease, and progressive disease. The primary endpoints were the correlation and agreement between post‐nCRT histopathologic‐ and MR‐based tumor staging. The secondary endpoints focused on predictive factors influencing the response to nCRT and assessing the effectiveness of MRI in identifying complete pathologic response (pCR).

**Results:**

The analysis showed a strong positive correlation (*r* = 0.86, *p* < 0.001) with moderate agreement (κ = 0.44) between post‐nCRT MR‐ and histopathologic‐based disease staging. According to multivariate analysis, initial tumor stage could predict clinical response to nCRT, and MR‐based TRG could predict histopathologic response. MRI exhibited a specificity of 96.2%, sensitivity of 22.2%, positive predictive value of 50%, and negative predictive value of 88.1% in detecting pCR.

**Conclusions:**

The study highlights the promising role of MRI in assessing treatment response in LARC, guiding clinical decision‐making and potentially reducing the need for invasive procedures to evaluate treatment effectiveness.

## 1 Introduction

Rectal cancer ranks as the eighth most prevalent malignancy globally [1], with locally advanced rectal cancer (LARC), which includes T3 and T4 tumors or tumors that affect regional lymph nodes, being identified in 5% to 10% of cases [2]. Neoadjuvant chemoradiotherapy (nCRT) has emerged as the standard treatment for LARC due to its potential to downstage the tumor, increasing the chances of successful surgical resection, and improving patient outcomes [3, 4].

While histopathologic restaging of the tumor after nCRT continues to be regarded as the gold standard for assessing treatment response, it has its limitations [5]. Histopathologic‐based tumor regression grade (TRG_HP_) is primarily ranked per the degree of fibrosis, tumor cellularity, and residual cancer. It is subjective and may yield inconsistent results between pathologists, leading to interobserver variability and potential discordance in treatment decision‐making [6]. An alternative approach relies on evaluating tumor response based on imaging findings. Application of diffusion‐weighted magnetic resonance imaging (DW‐MRI) for grading rectal tumor response is an area that continues to be extensively studied [7]. Knowledge of tumor response can help oncologists to escalate the neoadjuvant regimen to achieve a better tumor response or de‐escalate it to reduce the treatment toxicities and proceed the patient to earlier surgical resection. Hence, accurately identifying complete pathologic response (pCR) is crucial for informing therapeutic decisions [5]. DW‐MRI delivers information on tissue cellularity and vascularity, which facilitates the evaluation of treatment response and the prediction of pathological outcomes [8]. The ability to non‐invasively evaluate tumor response using MRI has the potential to address the limitations of histopathologic restaging and improve personalized treatment strategies for patients with rectal cancer. Besides, studies have introduced the MR‐based tumor response as a predictor of patients' survival [9].

Despite the growing use of MRI in restaging LARC after nCRT, there are still significant gaps in the literature. Limited data exist on the correlation between post‐nCRT MR‐based tumor regression grade (TRG_MR_) and the corresponding TRG_HP_. Some studies demonstrated a significant correlation [9, 10], while several others did not [11, 12]. The variation in findings may be attributed to the retrospective design of the studies in the current literature, which carries inherent limitations such as selection bias and insufficient control over confounding factors. Consequently, there is a need for prospective studies with stronger evidence to clarify and confirm this association.

To address these gaps, this prospective study aims to evaluate the correlation and agreement between post‐nCRT histopathologic‐ and MRI‐based tumor staging in LARC. Additionally, the study seeks to identify predictive factors influencing tumor response and assess the accuracy of MRI in detecting pCR. We hypothesize that the post‐nCRT MRI‐based tumor regression grade correlates strongly with the histopathologic tumor regression grade, serving as a reliable surrogate for treatment response assessment in LARC. Furthermore, we hypothesize that MRI‐reported tumor regression can predict the pathological tumor response to chemoradiation, facilitating more personalized and effective treatment planning. By providing robust evidence, this study aims to enhance the utility of MRI as a non‐invasive tool for restaging LARC, optimizing treatment strategies, and ultimately improving patient outcomes.

## 2 Materials and Methods

### 2.1 Ethical Confirmation

The experimental protocol for this study was in accordance with established regulations and guidelines, receiving confirmation from the Institutional Review Board of Iran University of Medical Sciences (IUMS). The study complied with the principles of the Declaration of Helsinki. The Ethical Committee of IUMS assigned the ethical code IR.IUMS.FMD.REC.1399.340. To promote transparency and meet reporting standards, this cohort study is reported based on Strengthening the Reporting of Observational Studies in Epidemiology (STROBE) checklist.

### 2.2 Study Design and Participants

This prospective cohort study examined previously untreated patients with histologically confirmed locally advanced rectal adenocarcinoma referred to the Oncology Departments of Shohadaye Haftome Tir and Firoozgar Hospitals in Tehran, Iran. Recruitment occurred between December 2019 and April 2022. Eligible participants were aged18–80 years, presenting with operable locally advanced disease, specifically Stage 2 or 3 disease without metastasis. Candidates were required to be in overall good health to endure the planned treatments, as determined by an ECOG (Eastern Cooperative Oncology Group) performance status of 0 or 1 [13]. Participants were required to provide written informed consent to enter the study; those who declined consent were excluded. Other exclusion criteria encompassed clinical indications of non‐regional lymph node involvement, recent diagnoses of inflammatory bowel disease or rheumatic conditions, compromised renal or hepatic function, and pregnancy or lactation. For calculating the sample size, the following formula was applied:

(1) $$n=\left[\left(Z_{\alpha }+Z_{\beta }\right)/0.5\times \ln\;{\left[\left(1+r\right)/\left(1-r\right)\right]}^{2}+3\right)$$

where, *Z*_*α*_ is the critical value corresponding to the desired Type 1 error rate (*α* = 0.01); *Z*_*β*_ is the critical value corresponding to the desired Type 2 error rate (*β* = 0.02), and *r* is the literature‐based correlation coefficient between two diagnostic modalities (*r* = 0.54) [14]. Based on the given values, the required sample size was 62 for this study.

### 2.3 Treatment and Assessment

Eligible patients underwent a standard clinical evaluation. Locoregional staging of the disease was conducted within 4 weeks prior to nCRT using contrast‐enhanced pelvic MRI, while assessment for distant metastasis was carried out through contrast‐enhanced computed tomography of the chest and abdomen, as deemed appropriate. The clinical stage was classified based on the eighth edition AJCC cancer staging [15]. The eligible participants received nCRT according to the long‐course protocol [16]. The treatment protocol involved long‐course chemoradiotherapy, which included radiation therapy targeting the entire pelvis at a total dose of 45 Gy, followed by a boost to the tumor bed with a 2 cm margin, culminating in a total prescribed dose of 50.4 Gy. Radiotherapy was delivered 5 days a week, with a daily dose of 1.8 Gy. During this period, patients also received oral capecitabine at a dose of 625 mg/m^2^ twice daily.

Throughout the treatment, patients were closely monitored with weekly assessments by physical examinations and routine laboratory tests to record the treatment‐related adverse effects. The concurrent administration of chemotherapy was stopped until the patient recovered in the following situations: when the absolute neutrophil count dropped below 1500/microliter, the platelet count decreased to less than 75 000/μl, or the patient experienced not manageable, grade 3–4 hand‐foot syndrome, vomiting, stomatitis, or diarrhea.

After 8–10 weeks of nCRT, patients underwent contrast‐enhanced pelvic MRI with a 1.5 Tesla MR device (GE HealthCare, The United States) under rectal protocol. An experienced radiologist reviewed the MR images of pre‐ and post‐nCRT in terms of TNM staging circumferential resection margin (CRM) status, and response to treatment. High‐resolution oblique axial T2‐weighted MR images were used to determine tumor volumes before and after chemoradiation therapy. This process involved delineating contours on each image slice to measure tumor cross‐sectional areas, which were then aggregated to calculate the total volume. Diffusion restriction was considered in diffusion‐weighted (DW) sequence to confirm the residual tumor. To minimize potential bias, the images taken at the time of disease diagnosis and after neoadjuvant therapy were anonymized to avoid affecting the radiologist's analysis. Within 10–12 weeks after neoadjuvant chemoradiotherapy (neoCRT), patients were planned to undergo total mesorectal excision (TME). The surgical approach aimed to achieve sphincter preservation whenever feasible.

### 2.4 Variables and Study Endpoints

The patients' baseline demographic (age, gender), laboratory (carcinoembryonic antigen [CEA]), and tumor data (tumor distance to the anal verge, tumor shape, and histologic grade) were collected before nCRT. MR‐associated parameters, including tumor and nodal stage, were gathered at baseline and within 8–10 weeks after the nCRT. The histopathologic response to nCRT was categorized according to the College of American Pathologists (CAP) consensus on TRG (Table S1) [17].

The study endpoints were to determine the correlation and agreement between post‐nCRT histopathologic‐ and MR‐based tumor staging (primary endpoint), the predictive factors of TRG_MR_ and TRG_HP_, and reporting the treatment toxicities (secondary endpoints). Additionally, the specificity, sensitivity, positive predictive value (PPV), and negative predictive value (NPV) of post‐nCRT MRI for detecting pCR were assessed. To identify potential predictive factors, clinical and demographic data of the patients were analyzed. A positive circumferential resection margin (CRM) was defined by the involvement of specific anatomical structures. For upper and mid‐rectal tumors, a positive CRM was indicated by direct tumor invasion of the mesorectal fascia or if the outermost margin of the tumor was within 1 mm of the mesorectal fascia. In the case of low rectal tumors, a positive CRM was identified if the tumor involved or was within 1 mm of the inter‐sphincteric plane or levator ani muscle [18]. The TRG_MR_ was classified into complete response (CR), partial response (PR), stable disease (SD), and progressive disease (PD) based on the RECIST (response evaluation criteria in solid tumors) criteria [19]. Treatment toxicities were assessed and categorized per Common Terminology Criteria for Adverse Events (CTCAE) version 4.0 [13].

### 2.5 Statistical Methods

All statistical analyses were conducted using IBM SPSS Statistics version 26. The Shapiro–Wilk test was applied to assess the normality of continuous data distributions. To assess the association between post‐nCRT MRI‐based and histopathologic TRG, we used Spearman's rank correlation coefficient (*r*). This non‐parametric test was selected due to the ordinal nature of TRG variables and the potential non‐normal distribution of scores. A correlation coefficient (*r*) ≥ 0.7 was interpreted as strong, 0.4–0.69 as moderate, < 0.4 as weak, and 0 as no correlation [20]. To evaluate the level of agreement between MRI‐based and histopathologic staging classifications, we used Cohen's kappa (*κ*) statistic, which quantifies agreement between categorical ratings beyond chance. *κ* values were interpreted as follows: < 0.20 = poor, 0.21–0.40 = fair, 0.41–0.60 = moderate, 0.61–0.80 = good, and > 0.80 = excellent agreement [21]. The diagnostic performance of MRI for identifying pCR was evaluated by calculating sensitivity, specificity, PPV, and NPV, using histopathologic findings as the reference standard. To explore predictive factors of clinical and histopathologic tumor response, we applied univariate logistic regression. A relaxed significance threshold of *p* < 0.25 was used in the univariate phase to prevent exclusion of potentially relevant variables. Variables with *p* < 0.25 were then entered into a multinomial logistic regression model to identify independent predictors of treatment response while adjusting for confounders. Group comparisons of categorical variables were analyzed using Chi‐squared or Fisher's Exact test, as appropriate. For comparisons of continuous variables that did not meet the normality assumption, the Kruskal–Wallis test was employed. A two‐sided *p*‐value < 0.05 was considered statistically significant for all final analyses, except where otherwise noted.

## 3 Results

### 3.1 General Characteristics

A total of 63 eligible participants were enrolled in this study. Table 1 represents a summary of the distribution of disease and patient characteristics. Of the eligible patients, 39 (61.9%) were male, and 36 (57.1%) were 60 or older. The age range was 20–80 years with a median age of 62 years. Regarding tobacco smoking history, most patients had no history of smoking (65.1%). Based on endoscopic findings, most tumors represented circumferential thickening (34.9%) or ulcerative lesions (28.6%). According to the WHO (World Health Organization) grading system, the tumors were almost well differentiated (55.6%) or moderately differentiated (41.3%). Clinical assessments indicated that the tumors were primarily at an advanced primary stage, with 85.7% of tumors graded as T3 or T4. Furthermore, 71.4% of patients exhibited regional lymph node involvement. Based on the AJCC classification, the majority of patients presented with stage III disease, comprising 73% of the study cohort. CRM was positive in 31 patients (49.2%).

**TABLE 1:** Baseline characteristics of participants.

Characteristics	Total (63 pts)
Gender	
Male, *n* (%)	39 (61.9)
Female, *n* (%)	24 (38.1)
Patient age at diagnosis (years)	
Mean ± SD	59.3 ± 14.0
20–39, *n* (%)	5 (7.9)
40–59, *n* (%)	22 (34.9)
60–80, *n* (%)	36 (57.1)
Tobacco smoking status^a^	
Positive, *n* (%)	22 (34.9)
Negative, *n* (%)	41 (65.1)
Tumor shape	
Ulcerative	18 (28.6)
Circumferential thickening	22 (34.9)
Polypoid growth	13 (20.6)
Obstructive	10 (15.9)
Tumor grade^b^	
G1 (well‐differentiated)	35 (55.6)
G2 (moderately differentiated)	26 (41.3)
G3 (poorly differentiated)	2 (3.2)
Tumor distance from AV (cm)	
Mean ± SD	7.1 ± 4.1
< 5, *n* (%)	24 (38.1)
5–10, *n* (%)	23 (36.5)
10–15, *n* (%)	16 (25.4)
Primary tumor stage	
T 2, 3, and T4	
*n*	9, 40, 14
%	14.2, 63.5, 22.2
Primary nodal stage	
N0, 1, and 2	
*n*	18, 20, 25
%	28.6, 31.7, 39.7
Primary clinical stage^c^	
I, *n* (%)	3 (4.8)
II, *n* (%)	14 (22.2)
III, *n* (%)	46 (73.0)
Circumferential resection margin	
Positive, *n* (%)	31 (49.2)
Negative, *n* (%)	32 (50.8)
CEA at diagnosis (ng/mL)	
Mean ± SD	10.9 ± 10.7

Abbreviations: AV, anal verge; CEA, carcinoembryonic antigen, nCRT, neoadjuvant chemoradiation; pts., patients; SD, standard deviation.

^a^ Current status at the time of entering the study.

^b^ Based on WHO grading system.

^c^ Based on AJCC 8th.

### 3.2 Clinical Response to Neoadjuvant Chemoradiation and the Predictive Factors

All patients completed the planned nCRT with manageable Grade 1 or 2 toxicities. Post‐neoadjuvant MR evaluation demonstrated CR in 4 (6.3%), PR in 31 (49.2%), SD in 24 (38.1%), and PD in 4 (6.3%). Logistic regression was conducted to explore the predictive factors TRG_MR_. Table 2 summarizes the univariate and multivariate results of the clinical response. The univariate analysis demonstrated that tumor shape, tumor stage, and AJCC staging were associated with the clinical response (*p* < 0.25). Tumors with circumferential thickening had lower clinical response rates than ulcerative, polypoid, and obstructive tumors (39.9% vs. 61.1%, 62.5%, and 70%, *p* = 0.22). The clinical response rate was significantly decreased with an increase in tumor stage (66.6% in T2, 57.5% in T3, and 42.8% in T4, *p* = 0.02). A similar pattern was recorded in categorizing patients based on AJCC staging (*p* = 0.15). The multinomial logistic regression analysis demonstrated the independent contribution of tumor stage (*p* = 0.04) to the clinical response to nCRT.

**TABLE 2:** Univariate and multivariate analysis of clinical response to neoadjuvant chemoradiation.

Characteristics	Clinical response, *n* (%)				*p* ^a^ (univariate)	*p* ^b^ (multivariate)
	CR (4 pts)	PR (31 pts)	SD (24 pts)	PD (4 pts)		
Gender					0.85	—
Male	3 (7.7)	18 (46.2)	15 (38.5)	3 (7.7)		
Female	1 (4.2)	13 (54.2)	9 (37.5)	1 (4.2)		
Patient age group					0.88^b^	—
20–39	0	2 (40.0)	3 (60.0)	0		
40–59	2 (9.1)	12 (54.5)	7 (31.8)	1 (4.5)		
60–80	2 (5.6)	17 (47.2)	14 (38.9)	3 (8.3)		
Tobacco smoking					0.29	—
Positive	3 (13.6)	9 (40.9)	8 (36.4)	2 (9.1)		
Negative	1 (2.4)	22 (53.7)	16 (39.0)	2 (4.9)		
Tumor shape					**0.22**	0.21
Ulcerative	0	11 (61.1)	7 (38.9)	0		
Circumferential thickening	2 (9.1)	7 (31.8)	11 (50.0)	2 (9.1)		
Polypoid growth	1 (7.7)	7 (53.8)	5 (38.5)	0		
Obstructive	1 (10.0)	6 (60.0)	1 (10.0)	2 (20.0)		
Tumor grade					0.73	—
G1	2 (5.7)	18 (51.4)	12 (34.3)	3 (8.6)		
G2‐3	2 (7.1)	13 (46.4)	12 (42.8)	1 (3.5)		
Distance from AV (cm)					0.57	—
< 5	1 (4.2)	9 (37.5)	11 (45.8)	3 (12.5)		
5–10	2 (8.7)	12 (52.2)	8 (34.8)	1 (4.3)		
10–15	1 (6.3)	10 (62.5)	5 (31.3)	0		
Tumor stage					**0.02**	**0.04**
T2	2 (22.2)	4 (44.4)	2 (22.2)	1 (11.1)		
T3	1 (2.5)	22 (55.0)	17 (42.5)	0		
T4	1 (7.1)	5 (35.7)	5 (35.7)	3 (21.4)		
Nodal status					0.25	—
N0	2 (11.1)	11 (61.1)	3 (16.7)	2 (11.1)		
N1	1 (5.0)	9 (45.0)	8 (40.0)	2 (10.0)		
N2	1 (4.0)	11 (44.0)	13 (52.0)	0		
Clinical stage					**0.15**	0.30
I	1 (33.3)	1 (33.3)	1 (33.3)	0		
II	1 (7.1)	9 (64.3)	2 (14.3)	2 (14.3)		
III	2 (4.3)	21 (45.7)	21 (45.7)	2 (4.3)		
CRM					0.79	—
Positive	1 (3.2)	16 (51.6)	12 (38.7)	2 (6.5)		
Negative	3 (9.4)	15 (46.9)	12 (37.5)	2 (6.3)		
CEA level (ng/mL)					0.98^c^	—
Mean (SD)	5.1 (4.6)	8.1 (9.5)	14.2 (10.3)	19.3 (32.4)		
Median	4.5	5.9	3.5	3.25		

Abbreviations: AV, anal verge; CRM, circumferential resection margin; SD, standard deviation.

^a^ The significant level is set to 0.25, based on the Chi‐Squared test for categorical variables and Kruskal–Wallis test for CEA results (in bold).

^b^ The significant level is set to 0.05, based on multinomial logistic regression (in bold).

### 3.3 Histopathologic Response to Neoadjuvant Chemoradiation and the Predictive Factors

All patients underwent TME with manageable post‐op complications. The histopathologic evaluation demonstrated TRG_0_ in 9 (14.3%), TRG_1_ in 10 (15.9%), TRG_2_ in 30 (47.6%), and TRG_3_ in 14 (22.2%). A logistic regression test was applied to find the predictive factors of histopathologic response to nCRT. Table 3 summarizes the univariate and multivariate results of the histopathologic response. The univariate analysis demonstrated that tumor grade, nodal status, AJCC staging, and MR‐based downstaging were associated with TRG_HP_ (*p* < 0.25). The rate of significant pathologic response (TRG 0, 1) was higher in low‐grade tumors compared with higher grades (37.2% in G1, 23.1% in G2, and none in G3, *p* = 0.10). The histopathologic response rate was significantly reduced with an increase in nodal involvement (TRG 0: 11.1% in N0, 15% in N1, and 16% in N2, *p* = 0.11). The multinomial logistic regression demonstrated that TRG_MR_ is the single significant predictor of the histopathologic response to nCRT (*p* = 0.004).

**TABLE 3:** Univariate and multivariate analysis of histopathologic response to neoadjuvant chemoradiation.

Characteristics	Histopathologic response, *n* (%)				*p* ^a^ (Univar.)	*p* ^b^ (Multivar.)
	TRG_0_ (9 pts)	TRG_1_ (10 pts)	TRG_2_ (30 pts)	TRG_3_ (14 pts)		
Gender					0.75	—
Male	5 (12.8)	5 (12.8)	19 (48.7)	10 (25.6)		
Female	4 (16.7)	5 (20.8)	11 (45.8)	4 (16.7)		
Patient age group					0.63	—
20–39	0	0	3 (60.0)	2 (40.0)		
40–59	4 (18.2)	3 (13.6)	12 (54.5)	3 (13.6)		
60–80	5 (13.9)	7 (19.4)	15 (41.7)	9 (25)		
Tobacco smoking					0.31	—
Positive	4 (18.2)	1 (4.5)	11 (50.0)	6 (27.3)		
Negative	5 (12.2)	9 (22.0)	19 (46.3)	8 (19.5)		
Tumor shape					0.59	—
Ulcerative	2 (11.1)	3 (16.7)	9 (50.0)	4 (22.2)		
Circumferential thickening	3 (13.6)	5 (22.7)	10 (45.5)	4 (18.2)		
Polypoid growth	4 (30.8)	1 (7.7)	6 (46.2)	2 (15.4)		
Obstructive	0	1 (10.0)	5 (50.0)	4 (40.0)		
Tumor grade					**0.10**	0.08
G1	5 (14.3)	8 (22.9)	17 (48.6)	5 (14.3)		
G2‐3	4 (14.2)	2 (7.1)	13 (46.4)	9 (32.1)		
Distance from AV (cm)					0.34	—
< 5	3 (12.5)	2 (8.3)	13 (54.2)	6 (25.0)		
5–10	4 (17.4)	7 (30.4)	8 (34.8)	4 (17.4)		
10–15	2 (12.5)	1 (6.3)	9 (56.3)	4 (25.0)		
Tumor stage					0.72	—
T2	1 (11.1)	2 (22.2)	5 (55.6)	1 (11.1)		
T3	7 (17.5)	5 (12.5)	17 (42.5)	11 (27.5)		
T4	1 (7.1)	3 (21.4)	8 (57.1)	2 (14.3)		
Nodal status					**0.11**	0.68
N0	2 (11.1)	5 (27.8)	11 (61.1)	0		
N1	3 (15.0)	1 (5.0)	9 (45.0)	7 (35.0)		
N2	4 (16.0)	4 (16.0)	10 (40.0)	7 (28.0)		
Clinical stage					**0.10**	0.46
I	0	1 (33.3)	2 (66.7)	0		
II	1 (7.1)	4 (28.6)	9 (64.3)	0		
III	8 (17.4)	5 (10.9)	19 (41.3)	14 (30.4)		
CRM					0.67	—
Positive	6 (19.4)	4 (12.9)	14 (45.2)	7 (22.6)		
Negative	3 (9.4)	6 (18.8)	16 (50.0)	7 (21.9)		
CEA level (ng/mL)					0.81	—
Mean (SD)	4.9 (3.6)	6.6 (9.8)	6.9 (7.5)	14.2 (26.9)		
Median	3.5	3.5	4.0	4.95		
MR‐based downstaging					**0.02**	**0.004**
CR	2 (50.0)	0	2 (50.0)	0		
PR	7 (22.6)	4 (12.9)	16 (51.6)	4 (12.9)		
SD	0	5 (20.8)	9 (37.5)	10 (41.7)		
PD	0	1 (25.0)	3 (75.0)	0		

Abbreviations: AV, anal verge; CR, complete response; CRM, circumferential resection margin; PD, progressive disease; PR, partial response; SD, stable disease and standard deviation; TRG, tumor regression grade.

^a^ The significant level is set to 0.25, based on the Chi‐Squared test for categorical variables and Kruskal–Wallis test for CEA results (in bold).

^b^ The significant level is set to 0.05, based on multinomial logistic regression (in bold).

### 3.4 Correlation and Agreement Between Histopathologic‐ and MR‐Based Tumor Staging

**FIGURE 1:**
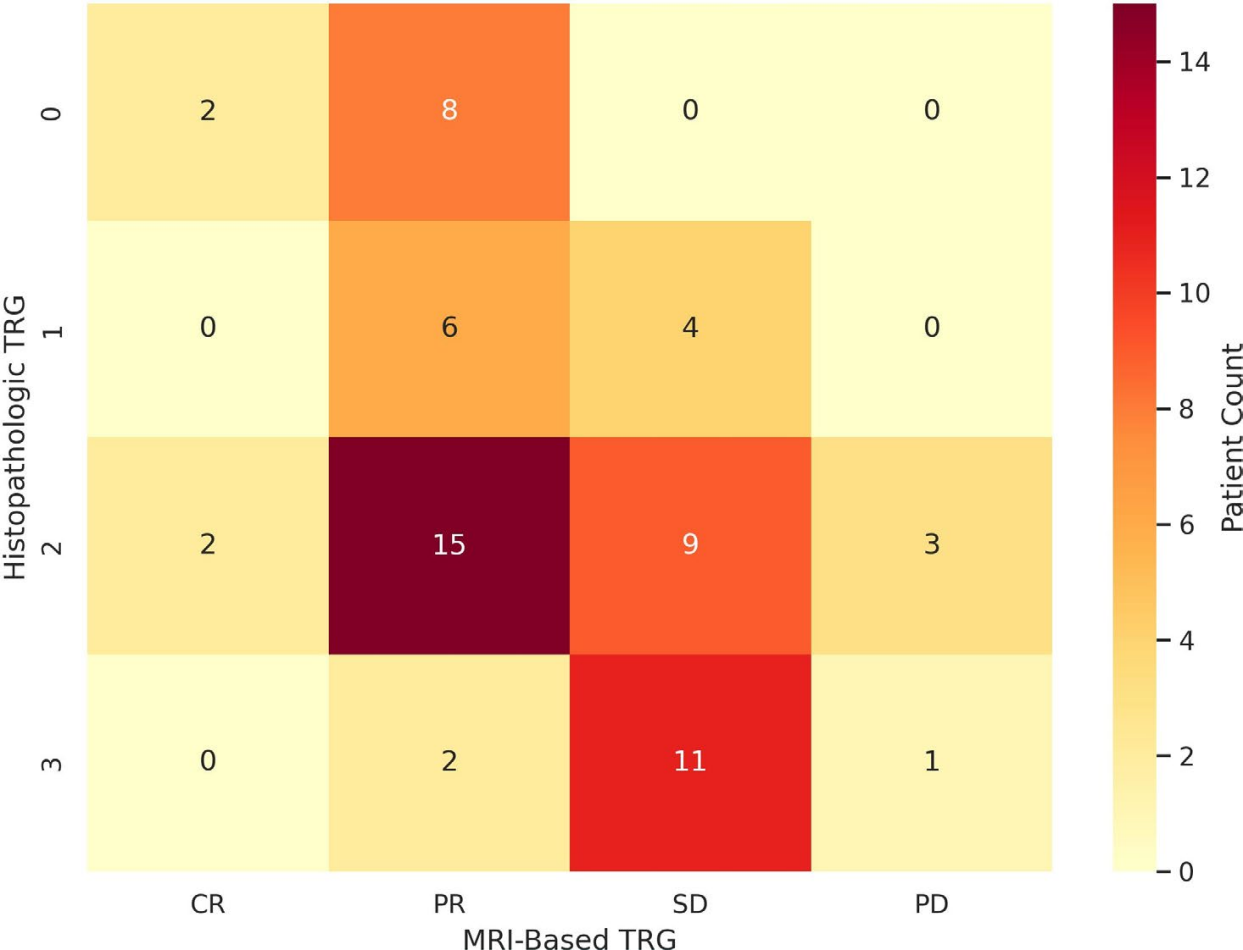
Distribution of patients across MRI‐based tumor regression grade (TRG‐MR: CR, PR, SD, PD) and histopathologic tumor regression grade (TRG‐HP: 0–3) following neoadjuvant chemoradiotherapy. Each cell indicates the number of patients in a given MR–HP TRG category pairing, with higher counts shaded more intensely. TRG indicates tumor regression grade.

**FIGURE 2:**
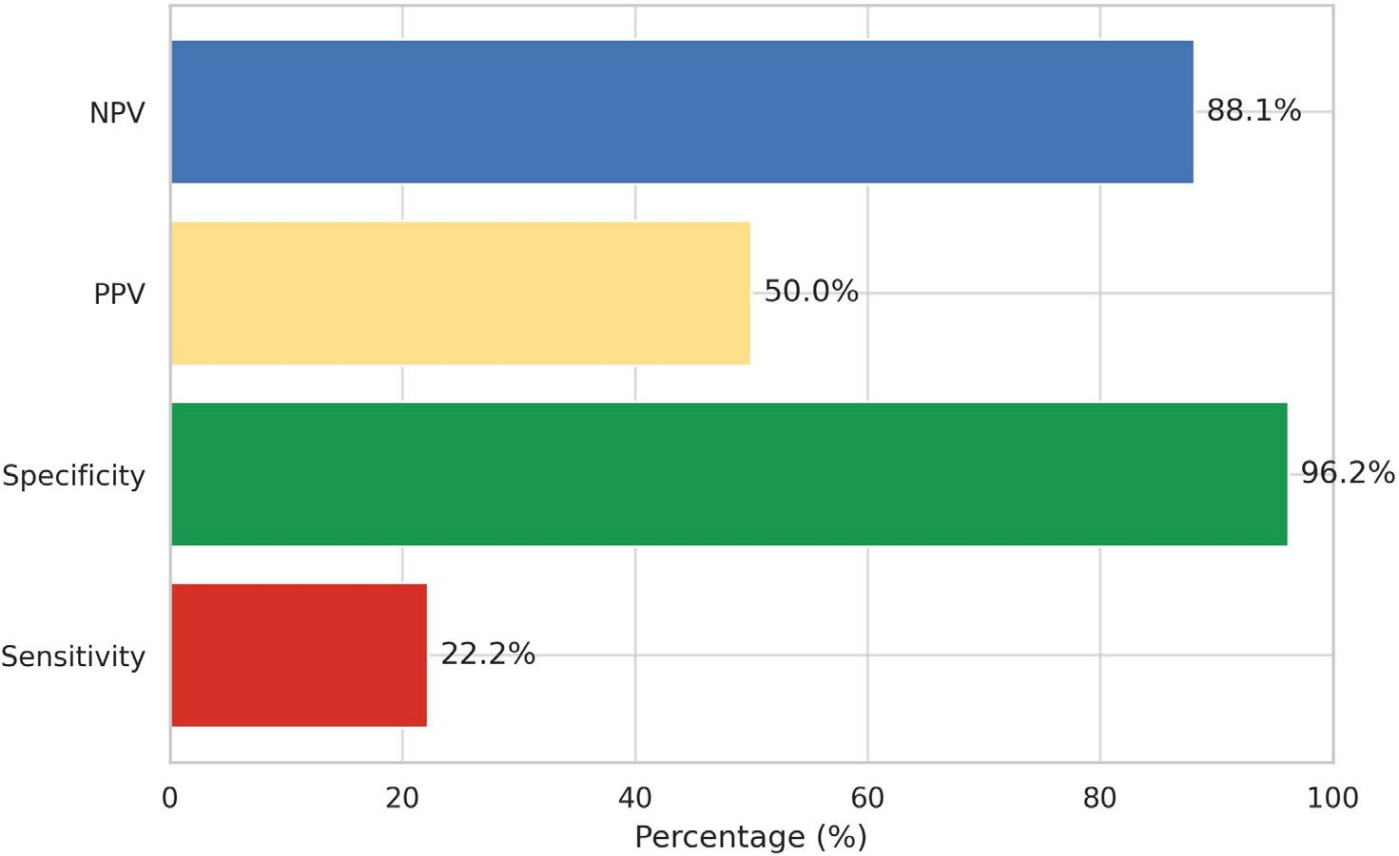
Diagnostic performance of MRI in detecting pathologic complete response. NPV indicates negative predictive value; PPV, positive predictive value.

A Spearman rank‐order correlation analysis was run to investigate the correlation between post‐neoadjuvant disease staging derived from MRI and histopathological findings. The results revealed a robust and statistically significant positive monotonic correlation between the two staging methods, with a correlation coefficient of *r* = 0.86 (*p* < 0.001). Subanalysis revealed a weak positive correlation between clinical (c) CR and pCR (*r* = 0.26, *p* = 0.03), a moderate positive correlation between cStage‐1 and pStage‐1 (*r* = 0.49, *p* < 0.001), no significant correlation between cStage‐2 and pStage‐2 (*r* = 0.19, *p* = 0.12), and a moderate positive correlation between cStage‐3 and pStage‐3 (*r* = 0.69, *p* < 0.001). Overall agreement between post‐nCRT histopathologic‐ and MR‐based tumor staging was 60.3% (*κ* = 0.44). Figure 1 illustrates the distribution of patients across corresponding MR‐based and histopathologic TRG categories, highlighting the overall alignment and points of discordance between the two grading systems. The specificity, sensitivity, PPV, and NPV of MRI to detect pCR were 96.2%, 22.2%, 50%, and 88.1%, respectively (Figure 2).

## 4 Discussion

This prospective cohort study was conducted on patients with LARC to determine the correlation between MR‐based tumor regression and histopathologic response to nCRT. The study indicated a strong positive correlation (*r* = 0.86) and moderate agreement (*κ* = 0.44) between post‐nCRT histopathologic‐ and MR‐based tumor staging. This study also demonstrated that MR‐based tumor regression is a significant predictive factor of histopathologic response to nCRT. In addition, we found that MRI has low PPV (50%) and high NPV (88%) in differentiating pCR in the resection specimens.

The significant correlation between TRG_MR_ and TRG_HP_ has also been demonstrated in previous studies. Popita et al. (2023) conducted a prospective study examining the correlation between MR‐based downstaging and pathology‐based downstaging of rectal cancer after nCRT. They reported a strong correlation (*r* = 0.73) between the two measures of downstaging [10]. In a separate study, Patel et al. indicated a moderate correlation between the two modalities (*r* = 0.65) [9]. In contrast, the literature contains studies indicating poor concordance between the two measures. For example, García‐Flórez et al. realized a weak correlation between the two diagnostic modalities in a retrospective cohort [12].

The current study also indicated moderate agreement between the two modalities. In line with this, Voogt et al. (2022) demonstrated moderate agreement (*κ* = 0.52) between TRG_MR_ and TRG_HP_ in patients with recurrent rectal cancer. The study demonstrated improved agreement with a shorter interval (< 7 weeks) between the end of nCRT and surgery [22]. In support, Patel et al. demonstrated moderate correlation between the two modalities (*κ* = 0.60) [9]. In a separate study, Rengo et al. (2017) found that applying a 3.0 Tesla scanner and automatic K‐Means clustering algorithm to detect post‐radiation fibrosis can enhance the agreement to 0.92 [23]. In addition, distinct TRG grading systems can show different agreement between MRI and histopathological results. For example, Sclafani et al. demonstrated fair agreement (*κ* = 0.24) between TRG_MR_ and five‐tier TRG_HP_ [11]. Therefore, one may conclude that the study setting, MR scanner sensitivity, and applied approach to detect and categorize the radiologic and pathologic response can affect the correlation outcomes. Future studies are invited to examine this notion.

This study also evaluated the diagnostic value of MRI in diagnosing pCR to nCRT. High NPV and low PPV of MRI in detecting pCR are consistent with a previous study conducted by Hall et al., which reported a PPV of 40% and an NPV of 84% for MRI in detecting pCR [24]. The consistent observation of low PPV and high NPV suggests that while MRI is fairly reliable in ruling out pCR, it has limitations in accurately confirming its presence. Given the high NPV (88.1%) observed in our study, MR TRG may play a pivotal role in guiding non‐operative management strategies, such as the ‘Watch and Wait’ approach. When integrated with digital rectal examination and endoscopic assessments, MR TRG could enable safe deferral of surgery in patients with a presumed clinical complete response, thereby reducing surgical morbidity [25]. Future studies incorporating multi‐modality assessments are warranted to optimize selection for such protocols.

Previous studies put forward certain pretreatment factors to enhance the probability of achieving a pCR upon nCRT. These factors consist of a low level of CEA at the time of diagnosis (< 5 ng/mL), a lower tumor grade, lower clinical T and N stages, a higher radiation dose, and a surgical delay of more than 6 to 8 weeks following preoperative treatment [26, 27, 28]. This study, however, did not demonstrate the predictive value of tumor grade, CEA, and initial clinical stage and found that MR‐based change in tumor volume is the single predictive factor of histopathologic tumor response. This controversy may be due to the retrospective design of those studies, which enfaces the results to the selection bias. Further prospective studies are required to determine the predictive factors of response to nCRT.

Our reported diagnostic performance of MRI in detecting pCR—specificity of 96.2%, sensitivity of 22.2%, PPV of 50%, and NPV of 88.1%—demonstrates a pattern of high specificity and NPV, but low sensitivity, which is consistent with previous literature. In a prospective study, Hall et al. reported a sensitivity of 68%, specificity of 62%, PPV of 40%, and NPV of 84% for MRI in predicting pCR following total neoadjuvant therapy in rectal cancer patients [24]. While their sensitivity was higher than ours, their specificity was notably lower, suggesting that MRI's ability to confidently exclude residual disease (high NPV) is more reliable than its capacity to confirm a complete response. Similarly, in a prospective cohort study of 50 patients, Maas et al. evaluated T2‐weighted MRI combined with diffusion‐weighted imaging (DWI) and found a sensitivity of 35% and a specificity of 94%, with an AUC of 0.79 (95% CI: 0.66–0.92) in detecting pCR [29]. These consistent findings support the notion that MRI is more effective in ruling out residual disease than in confirming a complete response, reinforcing the need for a multimodal assessment—including endoscopy and digital rectal examination—when considering watch and wait strategies.

The study findings need to be interpreted in light of the following limitations: (1) assessment of MR images by two radiologists and evaluating the agreement between their evaluations could help to improve the study's reliability. To address potential variability between radiologists in interpreting MRI results, several strategies can be implemented to enhance reliability and consistency. First, using specific validated guidelines for evaluating MR images ensures standardized assessment criteria across radiologists. Second, MR images can be independently reviewed by two experienced radiologists, with any discrepancies resolved through discussion to reach a consensus. In cases where disagreement persists, a third experienced radiologist can be consulted to provide a final decision. Third, radiologists should be blinded to the histopathologic response to eliminate bias in their evaluations; (2) the extramural vascular invasion was not included in the regression model predicting the pathologic response. The inclusion of this factor can further improve the study findings; (3) although the sample size was statistically justified based on prior correlation estimates, the relatively small cohort may limit generalizability and requires validation in larger, multicenter studies. In spite of these limitations, this is a handful of prospective research exploring the correlation between MR and histopathologic response to nCRT in patients with LARC, and the first evaluating this correlation in a society from Eastern countries.

This study can be a reliable reference for future cohorts and clinical trials. We acknowledge that genetic variations and tumor biology differences among diverse populations, such as Asian, African American, and Hispanic groups, may influence treatment response and toxicities [30, 31]. These factors could contribute to variability in the correlation between MRI‐based and histopathologic tumor staging. The current study is the first prospective analysis of this correlation in the MENA region, offering region‐specific data to help bridge this gap in oncologic imaging literature. To address this, future studies should validate the model in diverse populations, accounting for genetic and environmental factors, to enhance its generalizability and applicability across different demographic groups. Emerging studies have applied deep learning approaches, radiomics, and metabolic imaging (e.g., PET scan) to improve the diagnostic value of MRI in detecting the pathologic response to neoadjuvant treatments [32, 33, 34]. These innovative techniques aim to improve the accuracy and reliability of MRI‐based assessments, resulting in more precise evaluations of treatment responses and ultimately aiding in better patient management and decision‐making.

## 5 Conclusions

This study indicated a strong positive correlation and moderate agreement between the MR and histopathologic reports on rectal cancer response to nCRT. These findings reflect that MRI may be a surrogate for histopathologic response to nCRT. Besides, the study demonstrates that MRI can serve as a valuable modality to confirm the histopathologic complete response to nCRT. More research is required to confirm these results. This finding would facilitate the adoption of non‐operative management strategies and provide valuable prognostic information that complements histopathologic tumor response. The aim is to improve risk stratification and enhance post‐surgery patient outcomes through a more comprehensive approach.

## Author Contributions

Conceptualization: P.F. and K.N. Resources: P.F. and A.M.A. Data curation: M.G. and M.S. Writing original draft: S.S.R.N. and F.T.‐H. Final editing and validation: F.T.‐H. All authors have reviewed and given their approval for the final version of the manuscript.

## Ethics Statement

The Ethical Committee of Iran University of Medical Sciences approved the protocol of this study (Ethical code: IR.IUMS.FMD.REC.1399.340).

## Consent

The authors have nothing to report.

## Conflicts of Interest

The authors declare no conflicts of interest.

## Supporting information


**Table S1:** Guideline of College of American Pathologists on tumor regression grade.

## Data Availability

Data are available upon official request from the corresponding author.
