# Supplementary material for: Correlation Between MRI and Histopathology in Assessing Treatment Response to Neoadjuvant Chemoradiation in Locally Advanced Rectal Cancer
Source: Cancer Rep (Hoboken). 2025 Aug 28;8(9):e70322. doi: 10.1002/cnr2.70322 (PMC12394004; doi:10.1002/cnr2.70322)
Supplement: Supplementary file 1 — Table S1: Guideline of College of American Pathologists on tumor regression grade. [file CNR2-8-e70322-s001.docx]

| **Supplementary Table 1**. Guideline of College of American Pathologists on tumor regression grade | |
| --- | --- |
| CAP grading | Definition |
| TRG 0 | No viable tumor cells |
| TRG 1 | Single cells or small groups of tumor |
| TRG 2 | More residual cancer but overgrown by fibrosis |
| TRG 3 | Clear predominance of viable cancer with little or no signs of tumor regression |
